# The Analysis and Comparison of Anti‐Inflammatory and Antioxidant Characteristics of Postbiotic and Paraprobiotic Derived From Novel Native Probiotic Cocktail in DSS‐Induced Colitic Mice

**DOI:** 10.1002/fsn3.70034

**Published:** 2025-02-10

**Authors:** Niloofar Rezaie, Shadi Aghamohammad, Elham Haj Agha Gholizadeh Khiavi, Malihe Talebi, Mohammad Reza Pourshafie, Mahdi Rohani

**Affiliations:** ^1^ Department of Bacteriology Pasteur Institute of Iran Tehran Iran; ^2^ Department of Microbiology, School of Medicine Iran University of Medical Sciences Tehran Iran

**Keywords:** antioxidant agents, inflammatory bowel disease, oxidative damage, paraprobiotic, postbiotic

## Abstract

Oxidative stress, particularly when precipitated by the intake of a diet rich in fats, has the potential to induce an inflammatory state. Therefore, it is crucial to consider the administration of agents possessing antioxidant and anti‐inflammatory properties for the benefit of these patients. The objective of this study was to investigate the ability of postbiotic and paraprobiotic substances to regulate oxidative stress and inflammation. We hypothesized that both postbiotics and paraprobiotics could demonstrated significant efficacy in reducing oxidative stress and inflammation, with distinct differences in their effectiveness. A total of 88 *Lactobacillus* and *Bifidobacterium* strains were assessed for antioxidant activity. Male C57BL/6 mice were divided into four groups: HFD + PBS, HFD + DSS, HFD + DSS + postbiotic, and HFD + DSS + paraprobiotic. Various parameters, including weight change, disease activity index, and gene expression analysis, as well as enzymes involved in oxidative activities and inflammation were evaluated after treatment with derivatives of six selected strains. In comparison with the groups exposed to DSS, mice treated with a combination of postbiotic and paraprobiotic alongside DSS exhibited a reduction in DSS‐induced negative effects on both phenotypical characteristics and molecular indices, particularly the Nrf2‐ and NF‐kB‐related genes, with a notable focus on postbiotic. Based on the results, it can be inferred that despite the utilization of an unhealthy regime that may worsen oxidative stress and inflammation, the condition can be efficiently controlled by employing secure variations of probiotics, such as paraprobiotic and postbiotic components, with a particular emphasis on postbiotics.

## Graphical abstract

Oxidative stress from a high‐fat diet can lead to inflammatory conditions like inflammatory bowel disease (IBD), making it essential to explore agents with antioxidant and anti‐inflammatory properties. This study investigated the effects of postbiotic and paraprobiotic substances on oxidative stress and inflammation using 88 strains of *Lactobacillus* and *Bifidobacterium* in male C57BL/6 mice. Results showed that mice treated with postbiotics and paraprobiotics had reduced adverse effects from DSS, particularly regarding the expression of genes related to the NF‐kB and Nrf2 pathways, indicating that these agents can effectively manage oxidative stress and inflammation even in unhealthy dietary conditions.

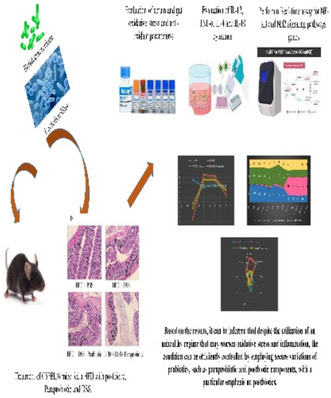

**Abbreviations**
CAT: catalase
CD: Crohn disease
DAI: Disease Activity Index
DSS: dextran sulfate sodium
GPX: glutathione peroxidase
GSH: glutathione
H&E: hematoxylin and eosin
HFD: high‐fat diet
IBD: inflammatory bowel disease
ISAPP: International Scientific Association for Probiotics and Prebiotics
Keap1: Kelch‐like ECH‐associated protein 1
MDA: malondialdehyde
NF‐Kb: nuclear factor‐kappa binding
ROS: reactive oxygen species
SLP: surface‐layer protein
SOD: superoxide dismutase
UC: ulcerative colitis

## 1 Introduction

Over the last decade, there has been a growing interest in probiotics, which are recognized as beneficial bacterial agents containing beneficial bacteria. In 2014, the definition of probiotics was established by the expert panel from the International Scientific Association for Probiotics and Prebiotics (ISAPP), led by Hill et al. According to their definition, probiotics are live microorganisms that, when consumed in appropriate quantities, provide a health advantage to the host (Martyniak et al. 2021). In addition to probiotics, the nonviable probiotic cell, known as paraprobiotic, and the byproducts referred to as postbiotic, are receiving significant attention due to their notable beneficial efficacies and high health benefit capacities (Vallejo‐Cordoba et al. 2020). According to the literature, postbiotics are consistent of byproducts including cell‐free supernatant, vitamins, organic acids, short‐chain fatty acids, secreted proteins/peptides, bacteriocins, and amino acids. These postbiotics have gained attention due to their known chemical structure, long storage stability, and their ability to activate various mechanisms in controlling inflammation, obesity, cancer, and oxidative stress (Nataraj et al. 2020). Paraprobiotic, which comprises of teichoic acids, muropeptides derived from peptidoglycan, molecules such as pili, fimbriae, and flagella, exopolysaccharides, proteins associated with the cell surface, and biosurfactants bound to the cell wall, assumes a vital role in the modulation of the immune response, suppression of cancer cell proliferation, alleviation of inflammation, inhibition of the growth of pathogenic bacteria, enhancement of the intestinal microbiome, and reduction of cholesterol levels (Nataraj et al. 2020; Akter, Park, and Jung 2020). Due to these advantageous characteristics and their inherent safety, these agents have drawn attention in recent years, especially for their ability to control inflammation and other detrimental processes in the body, including oxidative stress.

Oxidative stress, initially elucidated in 1985, denotes a phenomenon arising from the disarray between oxidant agents and antioxidant defense, which may potentially lead to biological impairment (Forman and Zhang 2021). Inflammatory bowel disease (IBD), including ulcerative colitis (UC) and Crohn's disease (CD) is a disorder that may arise as a result of oxidative stress. The connection between reactive oxygen species (ROS) and IBD is supported by scientific evidence, which suggests that higher levels of ROS combined with lower levels of antioxidants which play a significant role in causing IBD (Sahoo et al. 2023). The generation of ROS from inflammation plays a crucial role in the production of pro‐inflammatory cytokines, including IL‐1β, IL‐6, TNF‐α, and IFN‐γ. Furthermore, proinflammatory cytokines stimulate the accumulation of T cells and thereby promoting chronic inflammation. Thus, it could be said that the overproduction of ROS significantly contributes to the pathogenesis of IBD (Zeng et al. 2022). The situation in patients with IBD may worsen due to the consumption of a high‐fat diet (HFD), as this dietary regimen has the potential to trigger oxidative stress (Li et al. 2021) and contribute to an increased occurrence of inflammation. Henceforth, the utilization of any agent possessing antioxidant properties, including postbiotic and paraprobiotic, may prove to be essential in managing the symptoms present in patients diagnosed with IBD, particularly those individuals who are afflicted with obesity. The clinical investigations concerning the implications of probiotic byproducts have also demonstrated significant efficacy in patients suffering from IBD. According to numerous scholarly studies, short‐chain fatty acids, which constitute a prominent component of postbiotics, have the potential to reduce the Disease Activity Index (DAI) and may exhibit effectiveness comparable to mesalazine, a widely utilized pharmacological agent for the treatment of ulcerative colitis, Crohn's disease, and other forms of inflammatory bowel disease (Martyniak et al. 2021). Therefore, it seems that using the agents that could exert antioxidant and anti‐inflammatory effects could be useful for patients with inflammatory status.

Various signaling pathway are enrolled in oxidative stress. Nrf2, also known as nuclear factor erythroid 2‐related factor 2, is a transcription factor that responds to stress and is involved in maintaining cellular homeostasis. In normal situations, Nrf2 is localized in the cytoplasm due to the presence of Kelch‐like ECH‐associated protein 1 (Keap1), which aids in its degradation. However, when exposed to oxidative stress, Keap1 dissociates from the Nrf2/Keap1 complex, allowing Nrf2 to move into the nucleus. Once in the nucleus, Nrf2 activates the expression of various genes that are involved in antioxidation and inflammation reduction (Piotrowska et al. 2021; Hammad et al. 2023). Another signaling pathway that may be associated with oxidative stress is nuclear factor‐kappa binding (NF‐kB). NF‐kB is a protein complex that regulates the transcription of multiple genes. The NF‐κB pathway exhibits both antioxidant and pro‐oxidant functions in the context of oxidative stress (Moniruzzaman et al. 2018; Lingappan 2018). Thus, the utilization of an agent that has the potential to influence these signaling pathways may result in the manifestation of antioxidant properties, thereby rendering it a valuable tool in the management of IBD. As said above, postbiotic and paraprobiotic are beneficial derivatives of probiotic that could exert beneficial effects especially anti‐inflammatory and antioxidant properties. In this study, we aimed to investigate the antioxidant activities of our native postbiotic and paraprobiotic by examining their effects on the Nrf2 and NF‐kB signaling pathways. We also sought to determine which of these agents is more effective against oxidative stress in an in vivo model of colitis in mice. Indeed, we hypothesized that both the native postbiotic and paraprobiotic could significantly reduce oxidative stress in an In Vivo model of colitis by modulating the Nrf2 and NF‐kB signaling pathways.

## 2 Materials and Methods

### 2.1 The Phenotypic Assessment of Antioxidant Activity

Six different strains of probiotics were chosen based on their strong antioxidant properties, as determined by various biochemical tests such as DPPH, ABTS (Kim et al. 2022), superoxide anion (Xu et al. 2013), hydroxyl radical scavenging (Lin and Yen 1999), reducing power (Dilna et al. 2015), and lipid peroxidation inhibition assays (Ou, Ko, and Lin 2006). These strains include *Lactobacillus reuteri* 100, *Lactobacillus plantarum* 42, *Lactobacillus plantarum* 119, *Lactobacillus plantarum* 155, *Bifidobacterium bifidum* 1001, and *Bifidobacterium longum* 1044.

### 2.2 The Preparation of Postbiotic and Paraprobiotic

To produce postbiotic, 10^9^ cfu/mL of the probiotic strains that were obtained in our previous studies (Rohani et al. 2015; Eshaghi et al. 2017), such as *L. reuteri* 100, *L. plantarum* 42, *L. plantarum* 119, *L. plantarum* 155, *B. bifidum* 1001, and *B. longum* 1044, were subjected to centrifugation at a speed of 13,000 g for a duration of 5 min at a temperature of 4°C. Subsequently, the obtained supernatant was filtered using a 0.22 μm filter. The postbiotic cocktail was prepared by following the same method used for preparing individual strains.

Paraprobitics were obtained from the probiotic strains mentioned earlier. To generate paraprobitics, the probiotic strains were adjusted to a concentration of 10^9^ cfu/mL. Subsequently, the probiotic strains were heated at a temperature of 100°C for a duration of 10 min. The inactivated cells were then subjected to sonication at intervals of 10 min, with cycles lasting for 1 min and frequencies set at 40 Hz. This sonication process was carried out in an ice water bath specifically designed for sonication. The resulting suspension was further processed by centrifugation at a speed of 9000 g for 15 min, maintaining a temperature of 4°C. For the preparation of a paraprobiotic cocktail, all six selected strains (adjusted to a concentration of 10^9^ cfu/mL) were combined and subjected to the aforementioned method.

The assessment of the antioxidant activity of postbiotic and paraprobiotic was conducted by employing the aforementioned biochemical tests.

### 2.3 The Evaluation of the Antioxidant Activity in In Vivo Model

#### 2.3.1 Animal Maintenance

Twenty‐four male C57BL/6 mice, (4 to 6‐week‐old and 16 g), were procured from the Pasteur Institute of Iran. These mice were housed in groups of four in polycarbonate cages measuring 36 × 20 × 24 cm (length × breadth × wall height). They were maintained in a controlled environment with a temperature of 22°C and a humidity of 50%, following a regular light/dark cycle of 12 h of light and 12 h of darkness. The mice were received with standard food and water ad libitum for a period of 2 weeks. Then, the mice were put on an exclusive HFD consisting of 60% of total calories, with 35% from fat, 24% from protein, and 26% from carbohydrates, resulting in a caloric density of 52 kcal/g for a duration of 28 days. In the third week of the HFD regimen, when weight gain was observed, the HFD category was divided into four groups as follows: (1) HFD + PBS: A HFD in combination with 200 μL of PBS, (2) HFD + DSS: A HFD in combination with 200 μL of 2% dextran sulfate sodium (DSS), (3) HFD + DSS + Postbiotic: HFD in combination with 200 μL of a mixture containing 2% DSS and 10^9^ cfu/mL of our native postbiotic cocktail, and (4) HFD + DSS + Paraprobiotic: A HFD in combination with 200 μL of a mixture containing 2% DSS and 10^9^ cfu/mL of our native paraprobiotic cocktail.

#### 2.3.2 Histological Evaluation and the Analysis of Disease Activity Index

On the 14th day of experimentation, the mice were euthanized and their colons were collected for further analysis. The length of the colons served as an indirect indicator of inflammation. Subsequently, the colons were fixed in a 4% (w/v) solution of paraformaldehyde and prepared as multiple paraffin sections. Hematoxylin and eosin (H&E) staining technique was utilized, and the histopathological score was assessed in a blinded manner, adhering to the previously established protocols, including the characterization of crypt architecture, inflammation, muscle thickness, and goblet cell depletion (Ghanavati et al. 2020). According to Kwon et al. (2021), DAI scores were determined by taking into account factors such as weight loss, stool consistency, and bleeding. All experimental procedures were performed according to the ethical standards articulated in the Helsinki Declaration and received approval from the Animal Experimentation Committee of the Pasteur Institute of Iran (IR.PII.REC1400.061) for the ethical care and utilization of laboratory mice.

#### 2.3.3 Evaluation of Serum and Gut Oxidative Stress and Antioxidant Parameters

The level of MDA (Cat No. : NS‐15023), SOD (Cat No. : NS‐15033), CAT (Lot: Cat No. : NS‐15053), GSH (Cat No. : NS‐15087), GPX (Cat No. : NS‐15083) in both serum and distal intestinal tissue were evaluated in accordance with the instructions provided by the manufacturer (Navand Salamat, Iran).

#### 2.3.4 Evaluation of IL‐1β, TNF‐α, IL‐4, and IL‐10 Cytokines in Serum

The serum levels of IL‐1β, TNF‐α, IL‐4, and IL‐10 cytokines were measured with a high sensitivity double‐antibody ELISA test (Karmania Pars Gene, Iran), performed according to the manufacturer's instructions.

#### 2.3.5 Analysis of Nrf2‐ and NF‐kB‐Related Genes in the Colon by Quantitative Real‐Time PCR

Total RNA was isolated from homogenized mouse colon using the RNeasy Mini Kit (Favorgen Biotech Corp, Taiwan, Cat No. : 4, FAPRK 000‐Mini). cDNA synthesis kit was utilized to reverse transcribed of RNA (Yekta Tajhiz Azma Co, Iran, Cat No. : YT4500) and real‐time PCR was performed with 2x SYBR‐Green (RealQ Plus Master Mix Green, Amplicon A/S, Denmark, Cat No. : 4344463). The *gapdh*, as a housekeeping gene, was used to normalize the expression of the target gene. A list of primer sequences used in the current study is demonstrated in Table 1.

**TABLE 1:** Primers used in this study.

Genes	Primer sequence (5′ > 3′)	Product size
*Nrf2MF*	TAGATGACCATGAGTCGCTTGC	153 bp
*Nrf2MR*	GCCAAACTTGCTCCATGTCC	
*Keap1 MF*	TCGAAGGCATCCACCCTAAG	135 bp
*Keap1MR*	CTCGAACCACGCTGTCAATCT	
*NQO1MF*	AGGATGGGAGGTACTCGAATC	127 bp
*NQO1MR*	TGCTAGAGATGACTCGGAAGG	
*HO‐1MF*	GGTGATGGCTTCCTTGTACC	155 bp
*HO‐1MR*	AGTGAGGCCCATACCAGAAG	
*Trx‐1MF*	CTTTTGCCCGTCTCTCAATCA	181 bp
*Trx‐1MR*	AGGGTATTTCACACTTAGGTCCT	
*SOD2MF*	CAGACCTGCCTTACGACTATGG	113 bp
*SOD2MR*	CTCGGTGGCGTTGAGATTGTT	
*CATMF*	GGAGGCGGGAACCCAATAG	102 bp
*CATMR*	GTGTGCCATCTCGTCAGTGAA	
*Gpx1MF*	CCACCGTGTATGCCTTCTCC	105 bp
*Gpx1MR*	AGAGAGACGCGACATTCTCAAT	
*COX‐2 (PTGS2) MF*	TGCACTATGGTTACAAAAGCTGG	271 bp
*COX‐2 (PTGS2) MR*	TCAGGAAGCTCCTTATTTCCCTT	
*NF‐kBp65 (Rela) MF*	TGACCCCTGTCCTCTCACATCCG	94 bp
*NF‐kBp65 (Rela) MR*	CAGCTCCCAGAGTTCCGGTT	
*NF‐KBIA (IkBa)MF*	TGAAGGACGAGGAGTACGAGC	127 bp
*NF‐KBIA (IkBa)MR*	TGCAGGAACGAGTCTCCGT	
*Ikka (Chuk)MF*	GAGAGCGATGGTGCCATGAA	136 bp
*Ikka (Chuk)MR*	CCAGAACAGTACTCCATTGCCAGA	
*Ikkb (IKBKB)MF*	AAGTACACCGTGACCGTTGAC	91 bp
*Ikkb (IKBKB)MR*	GCTGCCAGTTAGGGAGGAA	
*GAPDHMF*	TGGCCTTCCGTGTTCCTAC	178 bp
*GAPDHMR*	GAGTTGCTGTTGAAGTCGCA	

### 2.4 Statistical Analysis

Graphs and statistical analysis of the data were performed using GraphPad Prism 8.0 (GraphPad Software Inc., United States) software to compare variables of different groups. Statistical differences between multiple groups were determined using ordinary one‐way ANOVA followed by Tukey's post hoc test, for data that showed normal distribution. *p* values < 0.05 were considered as statistically significant. The results were presented as standard deviation (SD).

## 3 Results

### 3.1 The Results of Biochemical Activity of Our Native Postbiotic and Paraprobiotic

The results of our biochemical assays are available in Table S1. An analysis of the biochemical tests revealed noteworthy findings pertaining to both our native postbiotic and paraprobiotic. The results indicated that our native postbiotic exhibited significantly higher antioxidant activities when compared to the paraprobiotics. The postbiotic with the lowest activity was found to be *B. longum* 1044, with an ABTS activity of 70.6, while the highest activity was observed in *L. plantarum* 119, with a DPPH activity of 81. On the other hand, the results for our native paraprobiotic demonstrated that *B. bifidum* RP1001 exhibited the lowest activity with regard to DPPH (46.3), while *L. reuteri* RP100 displayed the highest activity for Superoxide anion (72.6).

### 3.2 The Effects of Our Native Postbiotic and Paraprobiotic on DSS‐Induced Colitis

**FIGURE 1:**
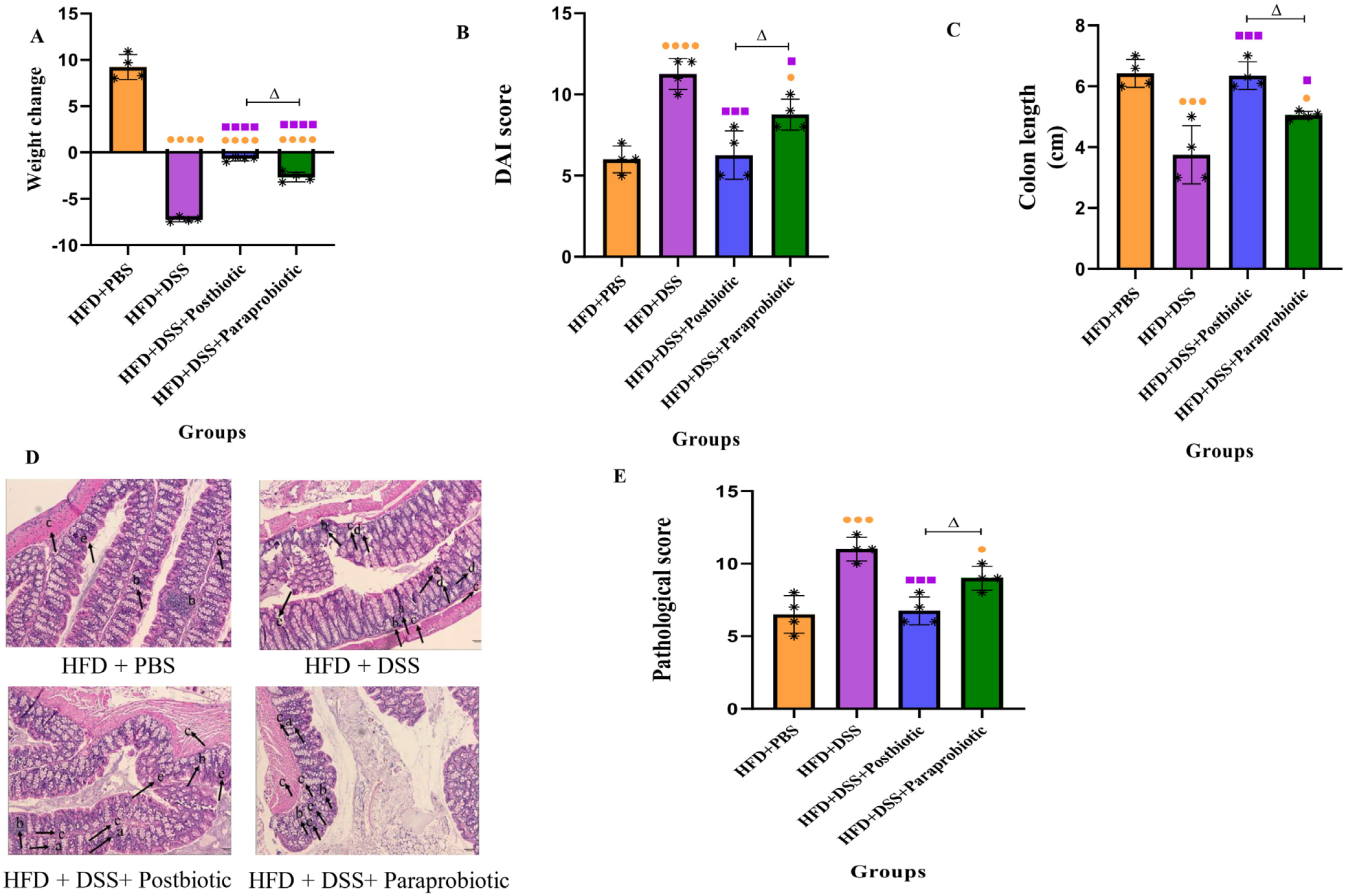
Effects of probiotics and postbiotics mixture on disease severity in DSS‐induced colitis mice. (A) Body weight changes, (B) DAI score, (C) colon length, (D) H&E staining of colon section of mice (a: Crypts architecture, b: Inflammation, c: Muscle thickness, d: Goblet cells depletion, and e: Crypts abscesses), the scale bar is 100 pixels. (E) histopathological score. Data are presented as the mean ± SD, *N* = 5 per group. Statistical significance was determined using the following symbols: Orange Circles: HFD + PBS vs. Other groups, Purple Square: HFD + DSS vs. Other groups, Triangle: The relatedness between HFD + DSS + postbiotic and HFD + DSS + paraprobiotic groups.

The anti‐inflammatory effects of our native postbiotic and paraprobiotic on mice with DSS‐induced colitis can be observed in Figure 1. Our findings indicate that the adoption of a high‐fat diet can lead to detrimental consequences in mice, such as obesity, reduced colon length, and increased DAI and pathological score, which demonstrate the adverse effects of inflammation in the gut. However, the use of our native postbiotic and paraprobiotic can certainly ameliorate the situation, as they are able to significantly reduce body weight (*p* < 0.05), DAI (*p* < 0.05), and pathological score (*p* < 0.001 for postbiotic), as well as increase colon length (*p* < 0.05). Furthermore, when comparing the postbiotic and paraprobiotic, the analysis of all colitis criteria reveals that our native postbiotic exhibits significantly greater anti‐inflammatory effects (*p* < 0.05). These agents can effectively mimic the status similar to the HFD + DSS, particularly with respect to DAI and pathological scores.

### 3.3 The Antioxidant Activity of Our Native Postbiotic and Paraprobitic in Serum and Gut

**FIGURE 2:**
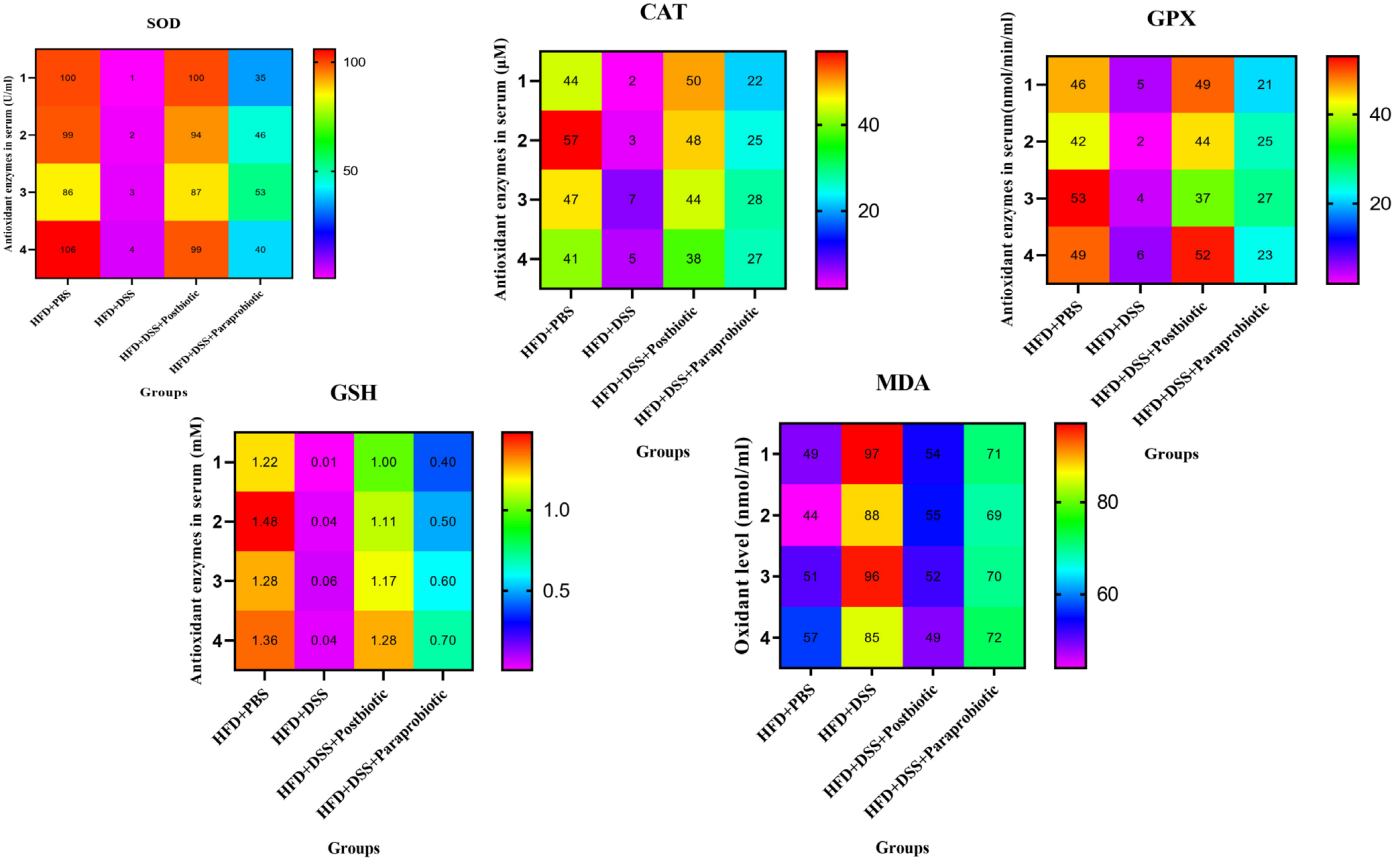
Levels of SOD, CAT, GSH, GPX (antioxidant enzymes) and MDA oxidant enzyme in serum. Data are presented as the mean ± SD, *N* = 5 per group. Statistical significance was determined using the following symbols: Orange Circles: HFD + PBS vs. Other groups, Purple Square: HFD + DSS vs. Other groups, Triangle: The relatedness between HFD + DSS + postbiotic and HFD + DSS + paraprobiotic groups.

**FIGURE 3:**
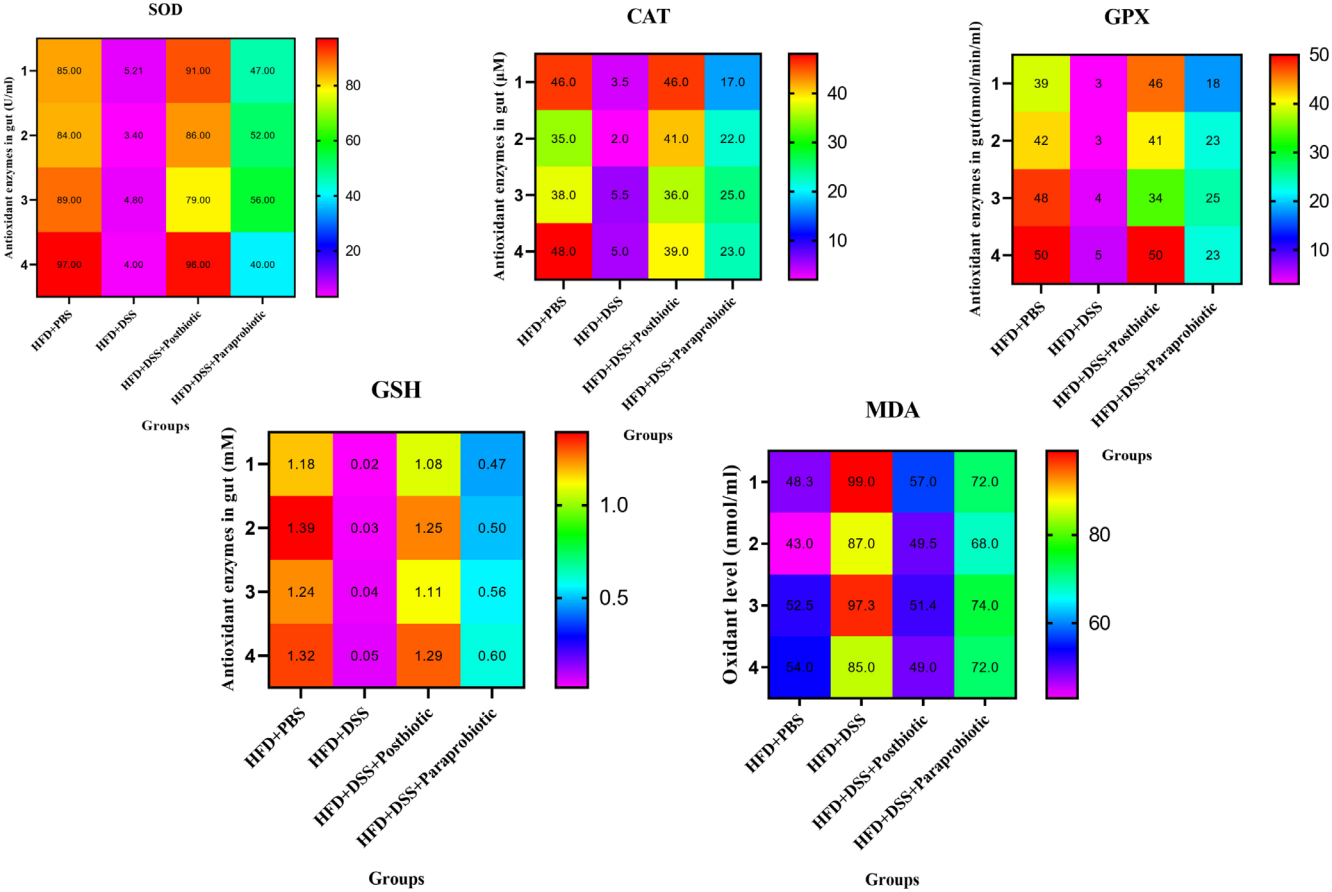
Levels of SOD, CAT, GSH, GPX (antioxidant enzymes) and MDA oxidant enzyme in gut. Data are presented as the mean ± SD, *N* = 5 per group. Statistical significance was determined using the following symbols: Orange Circles: HFD + PBS vs. Other groups, Purple Square: HFD + DSS vs. Other groups, Triangle: The relatedness between HFD + DSS + postbiotic and HFD + DSS + paraprobiotic groups.

The notable antioxidant activities of our native postbiotic and paraprobiotic are evident from the data presented in Figures 2 and 3. The heatmaps clearly demonstrate that the utilization of DSS resulted in a modification of color to red and yellow in relation to MDA, implying a significant augmentation in the degree of oxidant marker, as well as a change in color of markers SOD, CAT, GPX, and GSH to purple indicating the reduction of these antioxidant markers. Conversely, the administration of our native postbiotic and paraprobiotic induced a color change to light blue, green, and red, indicating a remarkable escalation in the levels of SOD, CAT, GPX, and GSH. In contrast, the dark blue color in MDA signifies a low level of oxidant marker. Importantly, the impact of postbiotics on all markers resembled that of the HDS + PBS group in terms of color change and was more pronounced compared to our native paraprobiotic agent. Interestingly, similar patterns were observed within the gastrointestinal tract, further highlighting the effectiveness and superiority of our native postbiotics.

### 3.4 The Anti‐inflammatory Properties of Our Native Postbiotic and Paraprobiotic Through the Modulation of IL‐1β, TNF‐α, IL‐4, and IL‐10 Cytokines in Serum

**FIGURE 4:**
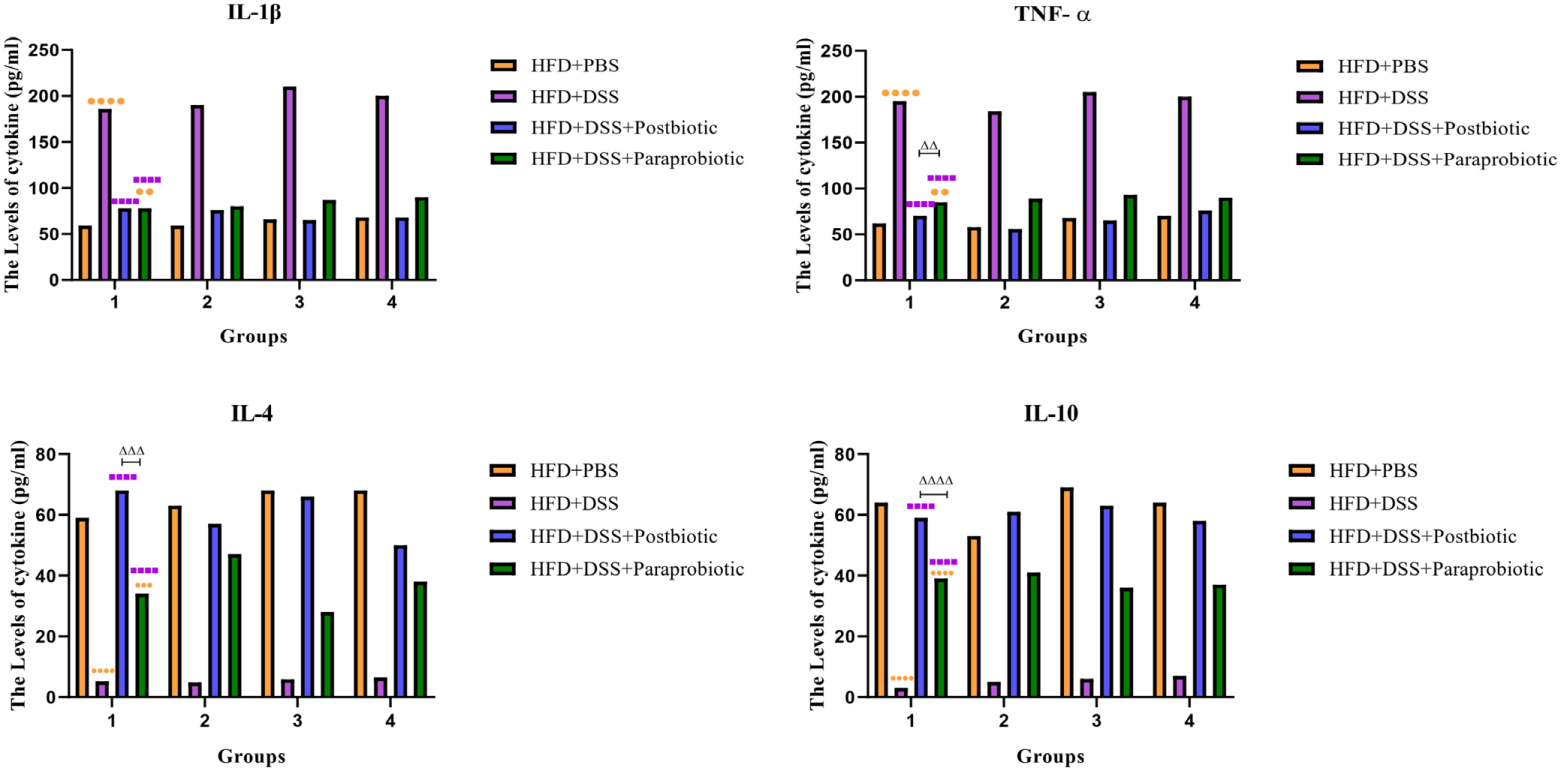
Levels of IL‐1β, TNF‐α (inflammatory cytokines) and IL‐4, IL‐10 (anti‐inflammatory cytokines), in serum. Data are presented as the mean ± SD, *N* = 5 per group. Statistical significance was determined using the following symbols: Orange Circles: HFD + PBS vs. Other groups, Purple Square: HFD + DSS vs. Other groups, Triangle: The relatedness between HFD + DSS + postbiotic and HFD + DSS + paraprobiotic groups.

The anti‐inflammatory effects of our native postbiotic and paraprobiotic again could be approved via analyzing the cytokine levels, as could be demonstrated in Figure 4. According to the findings of our study, DSS led to an increase in proinflammatory cytokines and a decrease in anti‐inflammatory cytokines (*p* < 0.0001), while the utilization of our native postbiotic and probiotic agents demonstrated a significant reduction in the levels of proinflammatory cytokines, including IL‐1β and TNF‐α (*p* < 0.0001). Conversely, the levels of anti‐inflammatory cytokines, including IL‐4 and IL‐10 were observed to increase significantly (*p* < 0.0001). In general, the use of our postbiotic agents exhibited notable more anti‐inflammatory effects regarding to TNF‐α, IL‐4, and IL‐10 (*p* < 0.01).

### 3.5 The Effects of Our Native Probiotic Strains on Gene Expression

**FIGURE 5:**
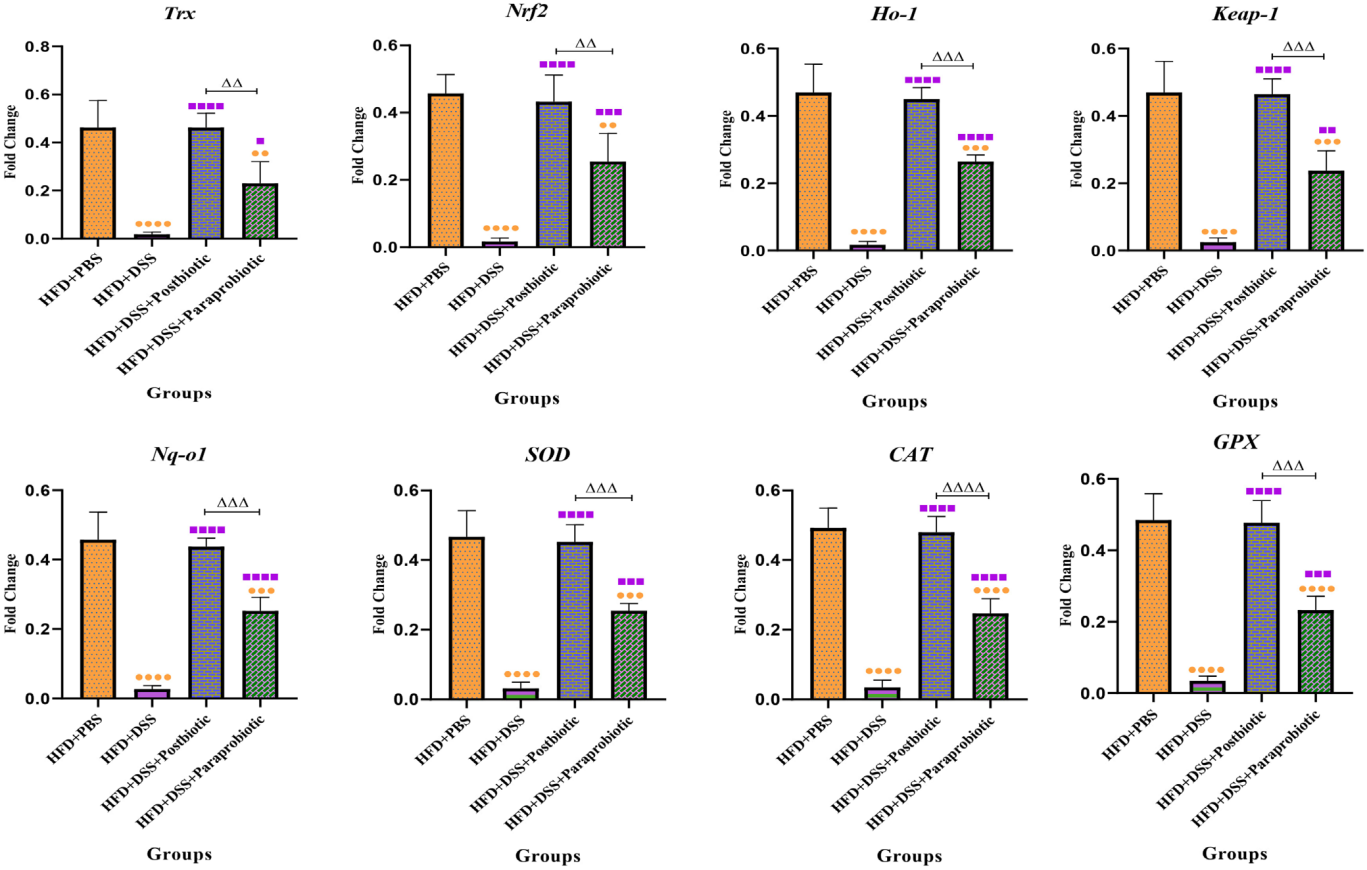
Relative gene expression (mean fold change) of antioxidants and Nrf2‐related pathway genes expression in the different groups of treatments. Data were normalized with *gapdh*. Data are presented as the mean ± SD, *N* = 5 per group. Statistical significance was determined using the following symbols: Orange Circles: HFD + PBS vs. Other groups, Purple Square: HFD + DSS vs. Other groups, Triangle: The relatedness between HFD + DSS+ postbiotic and HFD + DSS + paraprobiotic groups.

**FIGURE 6:**
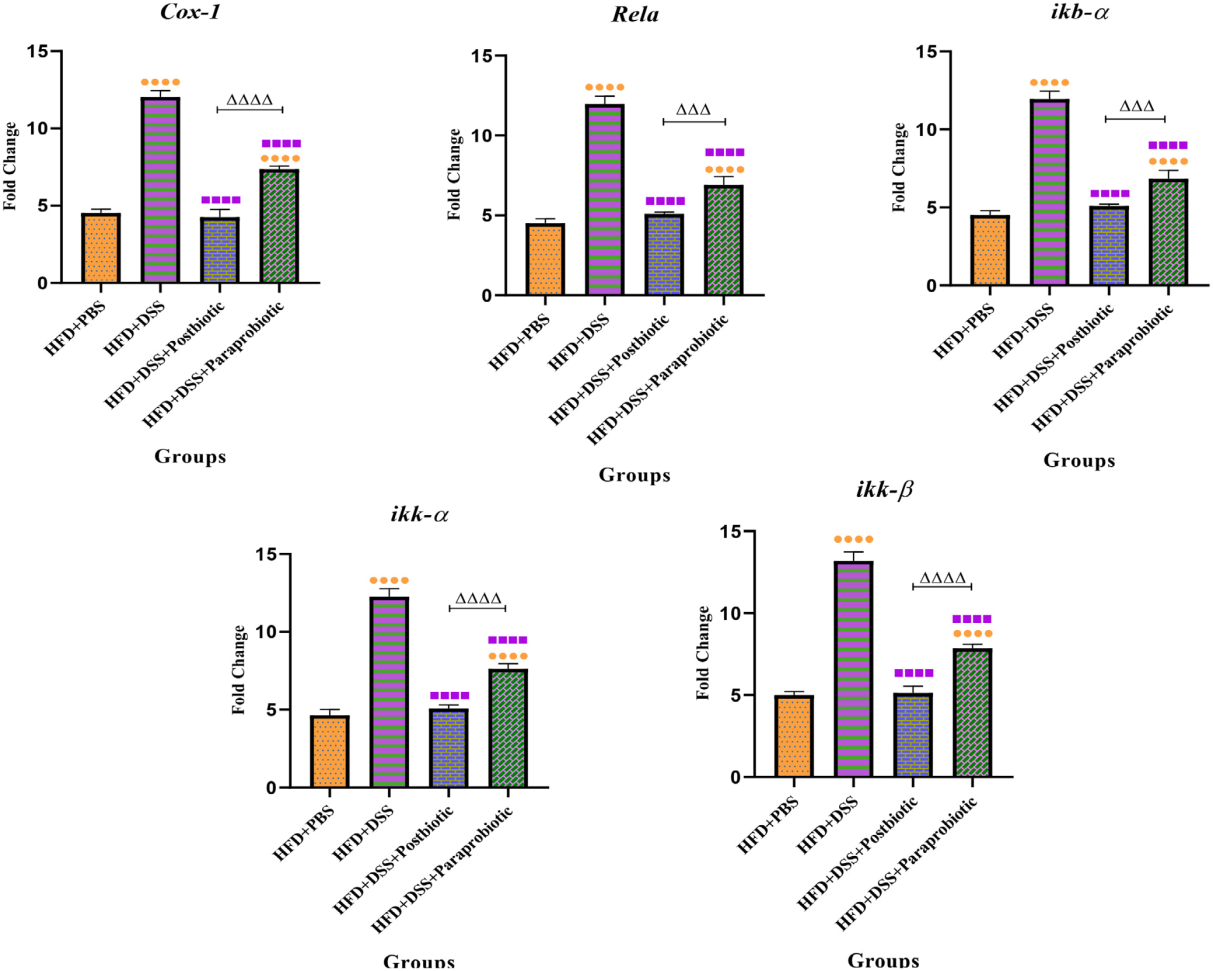
Relative gene expression (mean fold change) of NF‐kB‐related pathway genes expression in the different groups of treatments. Data were normalized with *gapdh*. Data are presented as the mean ± SD, *N* = 5 per group. Statistical significance was determined using the following symbols: Orange Circles: HFD + PBS vs. Other groups, Purple Square: HFD + DSS vs. Other groups, Triangle: The relatedness between HFD + DSS + postbiotic and HFD + DSS + paraprobiotic groups.

The impact of our native agents on the Nrf‐2 and NF‐kB signaling pathways can be observed in Figures 5 and 6. The utilization of DSS led to a decline in the expression levels of all Nrf‐2 genes (*p* < 0.0001) and an increase in the expression levels of genes associated with the NF‐kB pathway (*p* < 0.0001). Nonetheless, our native postbiotic and paraprobiotic strains were able to significantly augment the expression levels of Nrf‐2 (*p* < 0.05) and diminish the NF‐kB genes (*p* < 0.0001) in comparison with the DSS group. Once again, the postbiotic exhibited a more favorable effect (increase in Nrf‐2 level and decrease in NF‐kB) as compared to the paraprobiotic strains (*p* < 0.01). It can be concluded that our postbiotic was nearly capable of reinstating the gene expression level to that of the PBS + HFD group.

## 4 Discussion

The terms paraprobiotics, ghost probiotics, inactivated probiotics, and postbiotics highlight that the beneficial effects of bacteria do not rely on their viability. Inflammatory conditions like IBD may benefit from these agents, especially given the potential side effects of conventional treatments. Additionally, the rise of IBD in Western and industrialized countries is linked to high‐fat, low‐fiber diets, suggesting that probiotics and their byproducts could help mitigate inflammation and promote better health (Aggarwal et al. 2022; Teame et al. 2020; Tripathi and Feuerstein 2019; Andersen, Hansen, and Heitmann 2017). Here, our objective was to investigate the antioxidative and anti‐inflammatory properties of our native postbiotic and paraprobiotic substances. Additionally, we aimed to perform a comparative analysis to determine which agent is more effective in regulating oxidative stress and reducing inflammation.

**FIGURE 7:**
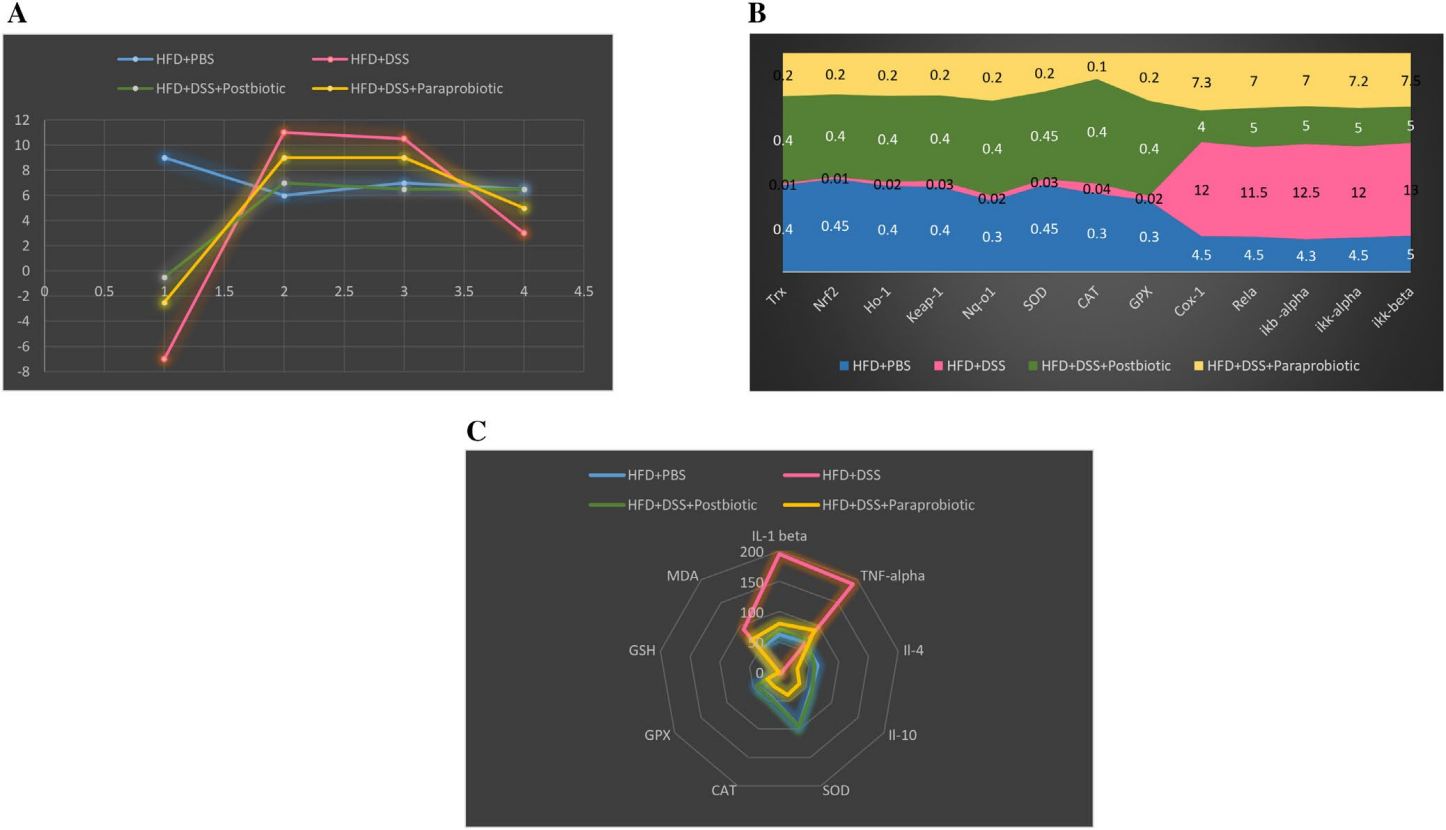
General trend of the current study. (A) The general trend of our native agents on pathological scores, including wight change, DAI, pathological scores, and colon length. (B) The general trend of our native agents on molecular pathways. (C) The general trend of our native agents on enzymes.

The general trend of the current study could be seen in Figure 7. Based on the phenotypic observations, both our postbiotic and paraprobiotic derivatives exhibit the potential to generate favorable outcomes in the production of antioxidant efficiencies in In Vitro assay, the antioxidant markers in serum and gut and also cytokine, in animal model with specific emphasis placed on the effectiveness of our postbiotics. Furthermore, our postbiotic demonstrates anti‐inflammatory effects in terms of its ability to prevent weight loss and mitigate other colitis indicators. It is anticipated that our mice will experience weight gain as a result of their consumption of a high‐fat diet for a period of approximately 28 days. Nevertheless, our derivatives have the capacity to minimize the degree of weight loss, with our postbiotic derivative even reducing it to nearly negligible levels, while also exerting an influence on the modulation of other colitis indicators in a manner comparable to the control group (HFD + PBS). There is a limited body of research that investigates the properties of postbiotics and paraprobiotics, and especially compares their potency in exerting anti‐inflammatory and antioxidant properties. In any case, our phenotypic findings align with the findings of other studies. According to Jang et al. (2018), the heat‐killed *L. plantarum* Ln1 could show the good antioxidant efficacies regarding to the reducing power and β‐carotene bleaching inhibitory activities tests. Aydin et al. (2021), observed that the postbiotics exhibited a DPPH free radical scavenging effect of up to 76.04%, whereas the paraprobiotic demonstrated an activity level of 18.07%. Consequently, the postbiotics displayed a greater degree of activity. Considering the effects of postbiotic and paraprobiotic in modulating the pro‐ and anti‐inflammatory cytokines, again our results are in accordance with others. Gao et al. (2017) reported that surface‐layer protein (SLP), DNA, exopolysaccharides, and CpG oligodeoxynucleotides extracted from *Lactobacillus rhamnosus* GG could exert anti‐inflammatory effects via reducing the mRNA expression of cytokines IL‐6, IL‐12, and TNF‐a in cells after LPS challenge. Shawky et al. (2023) found that by using heat‐inactivated *Bacillus subtilis* (HIB), they were able to increase the levels of superoxide dismutase, catalase, and glutathione peroxidase, while reducing the levels of malondialdehyde (MDA). These findings align with our own results.

As for the anti‐inflammatory and antioxidant properties of our native postbiotic and paraprobiotic, they exhibited significant effects on the regulation of Nrf2 and NF‐kB signaling pathways. These effects were observed in a manner that, as mentioned earlier, especially our postbiotic could potentially impact the expression level in a manner similar to the control group. Again, the quantity of research discussing the molecular antioxidant and anti‐inflammatory impacts of postbiotic and paraprobiotic substances is quite limited. Nonetheless, there exist a few studies that additionally document the effects of probiotic derivatives on the aforementioned signaling pathways. According to Nataraj et al. (2020), the surface proteins of *L. rhamnosus* have the ability to prevent the activation of the NF‐kB pathway which is caused by LPS. Additionally, it has been reported by Wang et al. (2020) that the lipoteichoic acid of *Akkermansia muciniphila* has the capacity to regulate the NF‐kB pathway, thereby enhancing the expression level of mucin (Muc2). In another study, Martyniak et al. (2021) reported that the activity of the NF‐κB complex is inhibited by butyrate in immune system cells. Consequently, the expression of genes that are responsible for the synthesis of the major proinflammatory cytokines, including TNF‐α, IL‐1β, IL‐2, IL‐6, IL‐8, and IL‐12 is decreased. Regarding Nrf2, it is worth noting that similar studies have demonstrated the advantageous effects of probiotic derivatives in exerting antioxidant activity. The work conducted by Karaca, Yilmaz, and Gursoy (2022) illustrates that diverse production of probiotics, such as exopolysaccharides derived from *L. rhamnosus* GG, have the ability to reduce H_2_O_2_‐induced oxidative damage in intestinal epithelial cells and enhance the antioxidant response by activating the Keap1/Nrf2 signaling pathways. Additionally, it has been observed that butyrate can stimulate Nrf2 through histone acetylation in the liver (Karaca, Yilmaz, and Gursoy 2022).

Hence, when considering the integration of this information, one could conclude that both postbiotics and paraprobiotics have advantageous properties in managing oxidative stress and inflammation, both in terms of physical characteristics and at a molecular level. In other words, it could be said that our research adds to the current knowledge base by showing that both postbiotics and paraprobiotics have notable antioxidant and anti‐inflammatory effects, consistent with earlier studies that emphasize the benefits of these substances in addressing issues like IBD. In particular, our findings reinforce previous investigations that demonstrate postbiotics have a greater antioxidant capacity than paraprobiotics, as indicated by their enhanced ability to alleviate oxidative stress and regulate cytokine levels. The biological processes that explain these findings may involve the influence of specific metabolites generated by postbiotics, including organic acids and proteins, which have been proven to bolster antioxidant defenses and diminish inflammation, supporting conclusions from recent research.

Actually, the beneficial effects attributable to postbiotics and paraprobiotics are of such a significant magnitude that these agents have the potential to be utilized in clinical applications. For instance, a randomized, double‐blind, placebo‐controlled trial was conducted to evaluate the effects of a postbiotic supplement comprising short‐chain fatty acids and exopolysaccharides derived from *Lactobacillus paracasei* , which revealed substantial reductions in high‐sensitivity C‐reactive protein (Hs‐CRP) levels. This finding suggests an anti‐inflammatory effect that may prove advantageous for patients in critical condition (Rahimi et al. 2024). The safety profile associated with postbiotics renders them compelling candidates for clinical investigations, particularly within vulnerable cohorts such as immunocompromised individuals, wherein traditional probiotics may present risks attributable to their live bacterial constituents (Avci, Yilmaz, and Avci 2024). In summary, the accumulating evidence derived from clinical trials substantiates the potential of postbiotics and paraprobiotics as innovative therapeutic agents capable of improving patient outcomes across a spectrum of medical conditions, thereby necessitating further investigation and validation through methodologically clinical research.

In addition to clinical trials, these substances may also yield positive outcomes in the fields of agriculture and industry. Abd El‐Ghany (2020) explore the potential of postbiotics and paraprobiotics as alternatives to antimicrobial growth promoters, which can enhance the growth and health of livestock and poultry, highlighting their immune‐supporting and growth‐promoting attributes. Additionally, Aggarwal explored the significance of postbiotics in the preservation of food, enhancing nutritional properties, and their applications in cosmetics, highlighting their versatility as functional elements across various sectors (Aggarwal et al. 2022).

The current results indicate that postbiotic and paraprobiotic treatments significantly alleviated DSS‐induced negative effects on both phenotypical characteristics and molecular indices, particularly concerning Nrf2‐ and NF‐kB‐related genes. This highlights the potential therapeutic benefits of these agents in managing oxidative stress and inflammation. By addressing oxidative stress and inflammation in the context of IBD, the study has significant implications for developing dietary interventions that could improve patient outcomes. However, there are also some limitations in the current study. The duration of treatment and observation in animal studies and also the used dosage of postbiotic and paraprobiotic may not fully capture the beneficial effects of postbiotics or paraprobiotics. Also, one significant limitation of protein evaluation using Western blotting is the reliance on the availability and specificity of primary antibodies of inflammatory cytokines.

## 5 Conclusion

According to the findings derived from the current investigation, it has been observed that various derivatives and products of probiotics, including postbiotics and paraprobiotics, possess the capability to exert a significant influence on the inhibition of oxidative stress as well as the display of anti‐inflammatory properties. Moreover, it is worth mentioning that the current study also sought to establish a comparison between postbiotics and paraprobiotics, with a particular emphasis on their respective anti‐inflammatory and antioxidant characteristics. The outcomes of this comparative analysis unequivocally demonstrated that postbiotics exhibited a more pronounced effect in this regard. It should be noted that the suppression of oxidative stress is widely acknowledged as a crucial element in maintaining one's overall health. This is particularly significant due to the prevalent adoption of diets that are rich in fat and lacking in fiber in modern dietary patterns, which could further intensify the occurrence of oxidative stress. Given these circumstances, it is highly reasonable to consider the administration of these safe agents as indispensable, particularly in the case of individuals afflicted with inflammatory conditions, including but not limited to inflammatory bowel disease. Given the promising results in reducing oxidative stress and inflammation, research should explore the development of functional foods enriched with special postbiotics and paraprobiotics. This could involve studying their stability, bioavailability, and sensory properties in food products, as well as conducting clinical trials to evaluate their efficacy in preventing or managing chronic diseases.

## Author Contributions

Performed the experiments: N.R.; Data analysis: S.A., N.R., and E.H.A.G.K.; Writing of the manuscript: S.A.; Revised manuscript: M.R., M.R.P., and M.T.; Conceived and designed the experiments: M.R. and M.T.

## Ethics Statement

The experimental protocols were established following the Declaration of Helsinki and approved by the ethics committee of Pasteur Institute of Iran (IR.PII.REC1400.061).

## Consent

Signed informed consent was obtained from all participants.

## Conflicts of Interest

The authors declare no conflicts of interest.

## Supporting information


Table S1.

## Data Availability

The datasets generated during and/or analyzed during the current study are available from the corresponding author upon reasonable request.
