# Supplementary material for: The Analysis and Comparison of Anti‐Inflammatory and Antioxidant Characteristics of Postbiotic and Paraprobiotic Derived From Novel Native Probiotic Cocktail in DSS‐Induced Colitic Mice
Source: Food Sci Nutr. 2025 Feb 10;13(2):e70034. doi: 10.1002/fsn3.70034 (PMC11808210; doi:10.1002/fsn3.70034)
Supplement: Supplementary file 1 — Table S1. [file FSN3-13-e70034-s001.docx]

Table S1. The antioxidant activity of our selected postbiotic

| Biochemical tests (Mean average%) | | | | | |  |
| --- | --- | --- | --- | --- | --- | --- |
| *Strains-post biotic cocktail* | DPPH | ABTS | HRS | Superoxide anion | RP | Lipid |
| *L. plantarum* 42 | 78±10.15 | 76.00±7.55 | 76.00±9.64 | 76.00±8.88 | 3.370±0.18 | 74.00±10.44 |
| *L. reuterri* 100 | 77±7.55 | 80±19.92 | 77.33±7.234 | 74.33±6.50 | 3.410±0.16 | 76.00±11.53 |
| *L. plantarum* 119 | 81±11.53 | 78.33±10.07 | 71.33±7.095 | 77±10.54 | 3.397±0.17 | 74±8.71 |
| *L. plantarum* 155 | 77±10.82 | 75±7.81 | 79.67±11.02 | 76.33±13.01 | 3.44±0.13 | 75±8.54 |
| *B. bifidum* 1001 | 79±7.55 | 75.33±9.07 | 73±15.13 | 79±10.54 | 3.340±0.05 | 72.67±3.21 |
| *B. longum* 1044 | 78.67±11.24 | 70.67±8.38 | 71.67±16.26 | 72±17.78 | 3.55±0.1 | 77.33±9.713 |
| postbiotic Cocktail | 83.67±11.59 | 82.67±6.5 | 84.33±5.68 | 86.33±6.65 | 3.65±0.06 | 84.33±9.86 |
| Positive control | 90.33±5.13 | 84.22±7.28 | 88.22±1.54 | 98.25±8.42 | .  3.630±0.35 | 90.67±17.47 |
| Negative control | 7.333±0.7 | 13.8±4.59 | 12±2 | 15.64±5.46 | 0.24±0.13 | 19.29±7.85 |

Table 3. The antioxidant activity of our selected paraprobiotic

| Biochemical tets (Mean average%) | | | | | |  |
| --- | --- | --- | --- | --- | --- | --- |
| Strains-paraprobiotic cocktail | DPPH | ABTS | HRS | Superoxide anion | RP | Lipid |
| *L. plantarum* RP42 | 57.67±6.11 | 64.33±12.66 | 66±5.56 | 70.33±10.69 | 2.880±0.08 | 59.33±8.38 |
| *L. reuteri* RP100 | 71.33±6.02 | 68.33±1.52 | 67.33±11.02 | 72.67±6.65 | 3.203±0.1 | 58.00±6.08 |
| *L. plantarum* 119 | 63±8.5 | 59.33±8.5 | 54.67±4.16 | 68.33±6.11 | 3.270±0.09 | 61.33±9.01 |
| *L. plantarum* RP155 | 65.67±51.32 | 69.67±5.68 | 48.67±4.09 | 51.33±5.5 | 3.007±0.09 | 50±5 |
| *B. bifidum* RP1001 | 46.33±8.73 | 55.67±5.13 | 48.00±6.08 | 60.67±8.96 | 2.893±0.12 | 59.67±9.01 |
| *B. longum* RP1044 | 52.33±7.76 | 51.33±2.3 | 59.33±4.61 | 66.67±8.08 | 2.760±0.14 | 55.67±19.01 |
| Paraprobiotic cocktail | 69±4.5 | 67.67±3.78 | 71.33±6.02 | 70.33±10.69 | 3.140±0.11 | 70.33±10.69 |
| Positive control | 90.33±5.13 | 84.22±7.28 | 88.22±1.54 | 98.25±8.42 | .  3.630±0.35 | 90.67±17.47 |
| Negative control | 7.333±0.7 | 13.8±4.59 | 12±2 | 15.64±5.46 | 0.24±0.13 | 19.29±7.85 |
